# Characterization of human papillomavirus type 16 pseudovirus containing histones

**DOI:** 10.1186/s12896-016-0296-3

**Published:** 2016-08-27

**Authors:** Hyoung Jin Kim, Hye-Lim Kwag, Hong-Jin Kim

**Affiliations:** Laboratory of Virology, College of Pharmacy, Chung-Ang University, 84 Heukseok-Ro, Dongjak-Gu, Seoul 06974 South Korea

**Keywords:** Human papillomavirus, Pseudovirion, Heparin chromatography, Histone

## Abstract

**Background:**

Pseudoviruses (PsVs) that encapsidate a reporter plasmid DNA have been used as surrogates for native human papillomavirus (HPV), whose continuous production is technically difficult. HPV PsVs have been designed to form capsids made up of the major capsid protein L1 and the minor capsid proteins L2. HPV PsVs have been produced in 293TT cells transfected with plasmid expressing L1 and L2 protein and plasmid containing the reporter gene. Several studies have suggested that naturally occurring HPV virions contain cellular histones, and histones have also been identified in mature HPV PsVs. However, the effect of the histones on the properties of the PsVs has not been investigated. Using heparin chromatography, we separated mature HPV type 16 PsVs into three fractions (I, II, and III) according to their heparin-binding affinities.

**Results:**

The amounts of cellular histone and cellular nucleotides per PsV were found to increase in the order fraction I, II and III. It appeared that PsVs in fraction I contains just small amount of cellular histone in Western blot analysis. The proportions of the three fractions in PsV preparations were 83.4, 7.5, and 9.1 % for fraction I, II, and III PsVs, respectively. In the electron microscope PsVs in fraction I appeared to have a more condensed structure than those in fractions II and III. Under the electron microscope fraction II and III PsVs appeared to be covered by substantial amounts of cellular histone while there was no visible histone covering PsVs of fraction I. Also the levels of reporter gene expression in infections of fraction II and III PsVs to 293TT cells were significantly lower than those in infections of fraction I PsV, and fraction II and III particles had significantly reduced immunogenicity.

**Conclusions:**

Our findings suggest that the involvement of large amounts of cellular histones during PsV formation interferes with the structural integrity of the PsVs and affects their immunogenicity. The fraction I particle therefore has the most suitable characteristics for use as an HPV PsV.

**Electronic supplementary material:**

The online version of this article (doi:10.1186/s12896-016-0296-3) contains supplementary material, which is available to authorized users.

## Background

Pseudoviruses (PsV) are synthetic viruses that can include genetic material such as DNA and RNA, and are closely related to the structures and characteristics of its native viruses, but lack characteristics shown in the authentic viruses such as capability for replication [1]. PsV systems permit the continuous production of virus particles mimicking naturally occurring particles, and provide high-throughput systems for evaluating anti-viral agents and vaccine candidates. Recently, various types of PsV systems have been developed. The PsV system for human immunodeficiency virus (HIV) uses the TZM-bI cell line, a genetically modified HeLa cell line expressing receptors for HIV and the firefly luciferase reporter gene under the control of the HIV long-terminal repeat [2]. PsVs for Merkel cell polyoma virus (MCV) have been generated by co-transfection with the VP1 and VP2 genes of MCV strain 339 and a Green fluorescent protein (GFP) reporter plasmid of 293TT cells engineered to stably express the cDNA of Simian virus 40 (SV40) T antigen [3]. Similar systems have been used for the PsVs of polyoma virus JCPyV [4], enterovirus 71 [5] and human papillomavirus (HPV) [6]. The HPV PsV system has evolved along with the success of the commercial vaccine against HPV. Currently the HPV PsV system is the most straightforward and widely used system in the HPV research field because the property of HPV PsV is similar to that of native HPV virion.

HPV is a non-enveloped DNA virus that infects cutaneous and mucosal epithelial tissues. Most cases of cervical cancer are caused by infection with high-risk HPV types [7, 8]. The HPV capsid is composed of 72 pentamers arranged on a T = 7 icosahedral lattice, each containing an L1 capsomeres [9, 10]. The capsid also includes the minor capsid protein L2 [11]. It has been suggested that the center of each capsomere is occluded with an L2 protein, and the L2 protein is located within the capsid [12, 13]. Some of the known roles of the L2 protein include facilitating capsid assembly, enhancing infectivity, and encapsidation of the HPV genome [14, 15].

Continuous production of the native HPV virion is difficult because its replication is strictly controlled by the cell cycle of the host cell [16, 17]. For this reason, synthetic HPV particles such as virus-like particles (VLPs), PsVs and quasivirions (QVs) have been substituted for native HPV virions in studies investigating aspects of infection, transmission, immunogenicity, and viral structure [18]. VLPs are composed of the L1 protein alone or L1 and L2 proteins [19–21], whereas the pseudovirus capsid contains both L1 and L2 proteins, and encapsidates reporter plasmid DNA [6]. QVs also contain L1 and L2 proteins, and encapsulate full-length HPV genomes [18, 22]. Capsid structure is thought to be stabilized by intermolecular disulfide bonds between capsomeres: an analysis of recombinant HPV16 VLPs suggested that a critical intercapsomeric disulfide bridge occurs between Cys428 and Cys175 [9, 10, 14]. In the case of synthetic HPV virions such as PsVs, and unlike in other viruses, a long period of maturation (>24 h at 37 °C) is required because the formation of disulfide bonds is slow [14].

The HPV genome is approximately 8 kb in length and is replicated in the nucleus [18]. The genome of native HPV virions is thought to have a chromatin-like structure that includes host histones [23], and cellular histones are also thought to be present in the mature HPV PsVs [13]. The presence of cellular histones has been found to be associated with reduced infectivity of HPV virions and of infectious PsVs. However, the properties of the histone-carrying PsVs have not been investigated in detail.

It is generally agreed that differences in the heparin binding affinities of different viruses that attach to the cell via heparin sulfate are responsible for differences in their properties, such as virulence and pathogenesis [24–29]. Since cellular histones bind strongly to heparin [30], we hypothesized that the histone-containing HPV PsVs would bind heparin more strongly than those without histones. In the present work we identified three types of HPV type 16 (HPV16) PsVs according to their heparin-binding affinities, and compared their structures, infectivity and immunogenicity, in order to identify the specific characteristics of histone-carrying PsVs.

## Results

### Separation of HPV16 PsVs by heparin chromatography

**Fig. 1:**
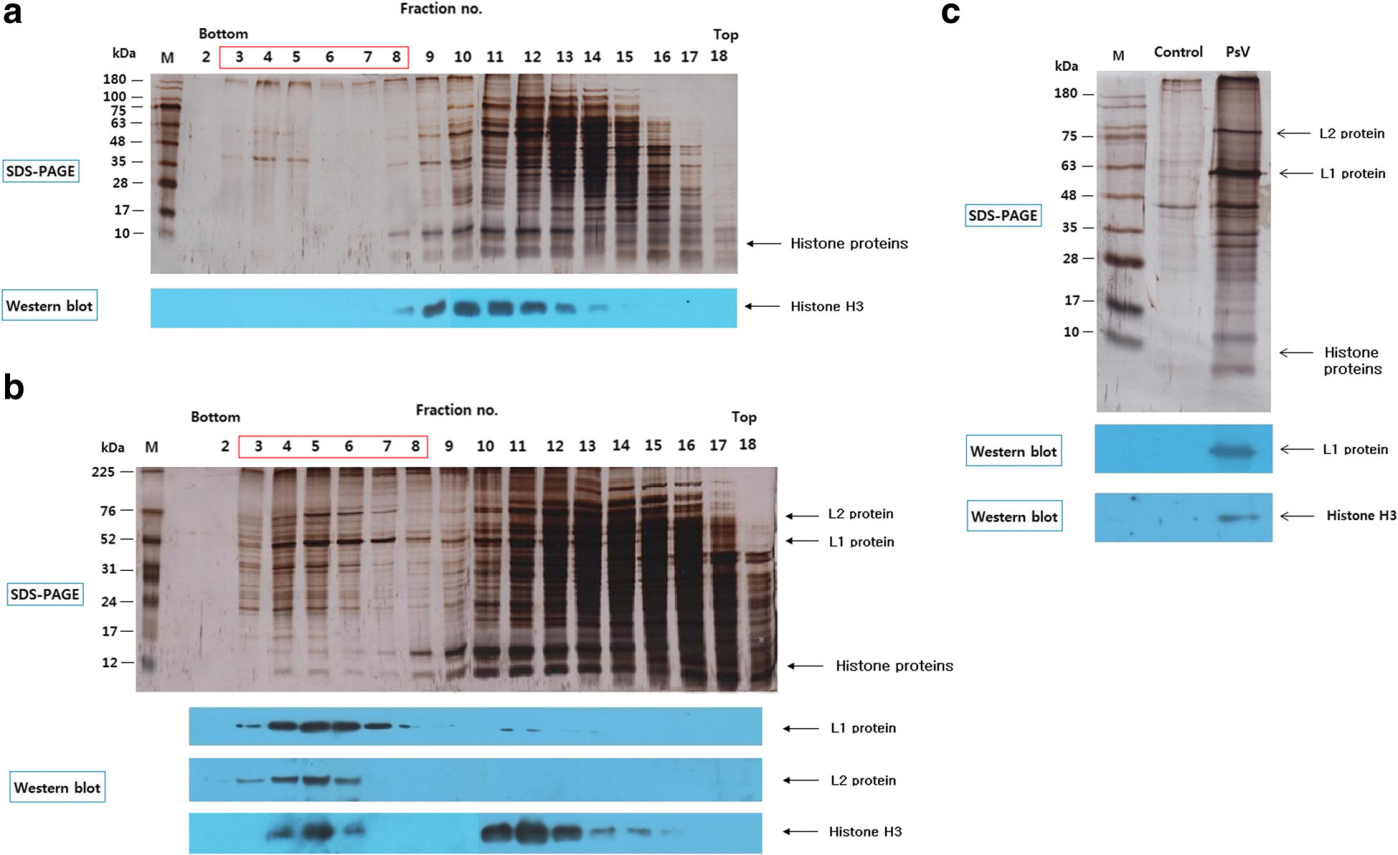
Purification of mature HPV16 PsVs by SEC. **a** SDS-PAGE and Western blots for human histone H3 of SEC fractions of a 293TT cell lysate (control experiment). **b** SDS-PAGE and Western blots for detecting L1, L2 and human histone H3 of SEC fractions of a lysates of 293TT cells transfected with p16sheLL and pYSEAP. **c** SDS-PAGE and Western blots for detecting L1 protein and human histone H3. Control refers to a mixture of fractions 3 – 8 in panel **a**; PsV refers to a mixture of fractions 3 – 8 in panel **b**. M is a protein marker. PsVs eluted early (in fractions 3 to 8, *red box* in panel **b**), whereas most cellular proteins eluted later (fractions 10 to 16), indicating that the PsVs were larger than most of the latter (**b**). Histone H3 co-eluted with L1 protein in fractions 4 to 6 (**b**) and there was no histone H3 in the corresponding fractions of the 293TT cell control (**a**). Fractions 3–8 from the 293TT cell control were combined, as were fractions 3–8 from the 293TT cells producing HPV16 PsVs (*red boxes* in **a** and **b**); they were then analyzed by SDS-PAGE and Western blotting (**c**)

Mature HPV16 PsVs were produced in 293TT cells by co-transfection of p16sheLL (expressing L1 and L2 proteins) and pYSEAP (expressing SEAP) and purified by size-exclusion chromatography (SEC, Fig. 1), and the PsVs were subdivided into fractions I, II, and III according to their heparin-binding affinities (Additional file 1: Figure S1 and Additional file 2: Figure S2): the heparin chromatography condition facilitates separation of PsVs containing small amounts of cellular histone from those with large amounts of histone. SEC demonstrated that cellular histones are principally involved in the production of HPV PsV (Fig. 1). It is known that correctly folded HPV capsids and HPV PsVs can bind to heparin-bound resin in 0.325 M NaCl at pH 7.2 [31, 32]. In the present work 0.65 M NaCl at pH 7.2 was used for the binding studies because under these conditions HPV PsVs bind only when they contain substantial amounts of histone (Additional file 1: Figure S1 and Additional file 2: Figure S2). Thus the flow-through fraction under these conditions was designated fraction I PsV. The PsVs bound to the heparin were eluted successively with buffer containing 0.8 M NaCl and buffer containing 1 M NaCl, and the eluted PsVs were designated fraction II and III PsVs, respectively (Additional file 1: Figure S1). The detail sodium dodecyl sulfate-polyacrylamide gel electrophoresis (SDS-PAGE) banding patterns of the fractions from the heparin chromatography can be seen in Additional file 2: Figure S2.

### Comparison of histone and cellular DNA contents

**Fig. 2:**
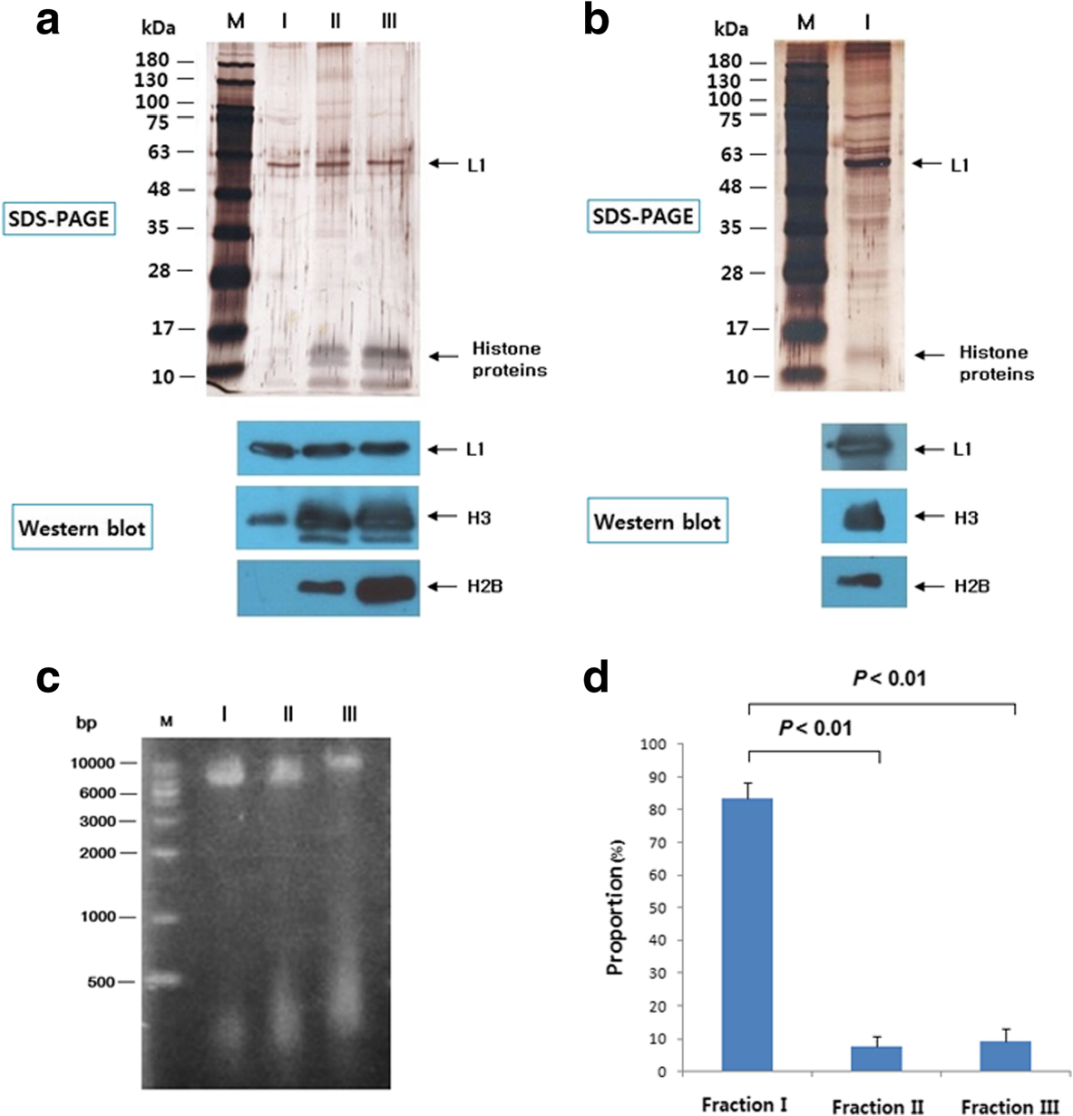
Analysis of histones, cellular DNA contents and proportions of HPV16 PsVs from fraction I, II and III. **a** shows SDS-PAGE and Western blots comparing the L1, histone H3 and histone H2B contents of the three types of PsVs. **b** shows SDS-PAGE and Western blots for detecting L1, histone H3 and histone H2B in type I PsVs. **b** is a repeat of panel A but with extended film exposure for Western blots. **c** is a result of agarose gel showing cellular DNA contents of PsVs from fraction I, II and III. **d** is result showing the proportions of PsVs from fraction I, II and III. To calculate the proportion of each type, their L1 content was determined by sandwich ELISA. Data represent the mean ± SD of four independent experiments

To compare the histone H3 and H2B contents of the three PsV types, identical amounts of PsVs (based on L1 protein content) were loaded (Fig. 2a). It was found that the virus-associated contents of histone protein increased in the order fractions I, II and III (Fig. 2a). PsVs in fraction I appeared to contain small amounts of histones H3 and H2B, which could be seen when the antibody reaction and film exposure times were extended (Fig. 2b). It was confirmed that the amounts of cellular DNAs less than 1000 bp contained in the three PsVs paralleled the amounts of histones (Fig. 2c). The L1 protein content of each fraction of PsV was determined by sandwich enzyme-linked immunosorbent assay (ELISA) (Fig. 2d), and the proportions of PsVs from fraction I, II, and III inferred from this were 83.4, 7.5, and 9.1 %, respectively (Fig. 2d). Therefore, PsV in fraction I, which contains only a small amount of cellular histone, appears to be the predominant type.

### Comparison of the structural integrity of HPV16 PsVs from fraction I, II and III

**Fig. 3:**
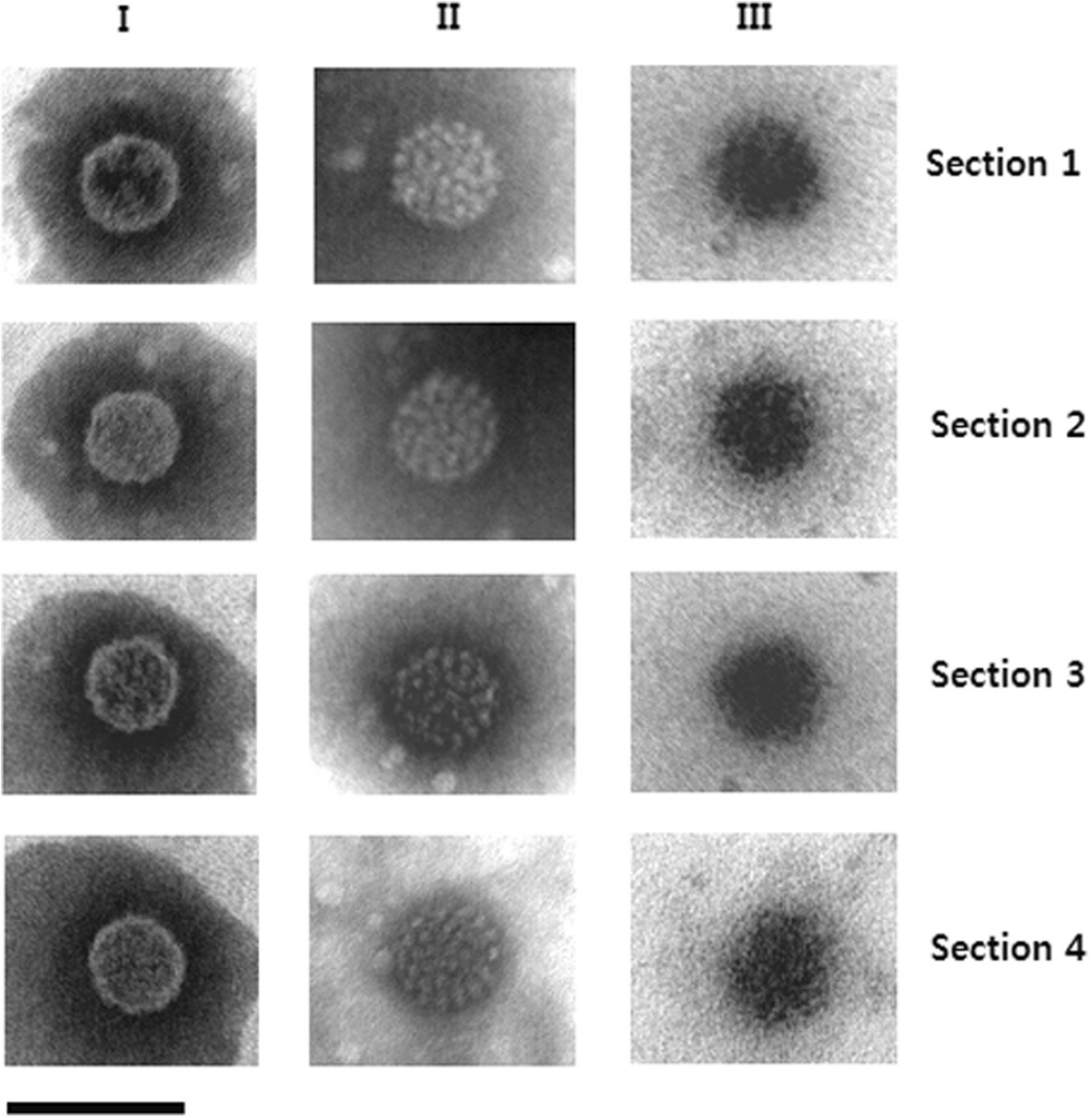
TEM analysis of HPV16 PsVs of fractions I, II and III. The presented images were representatives from triple observations. Magnification is 234,000× (bar 100 nm)

**Fig. 4:**
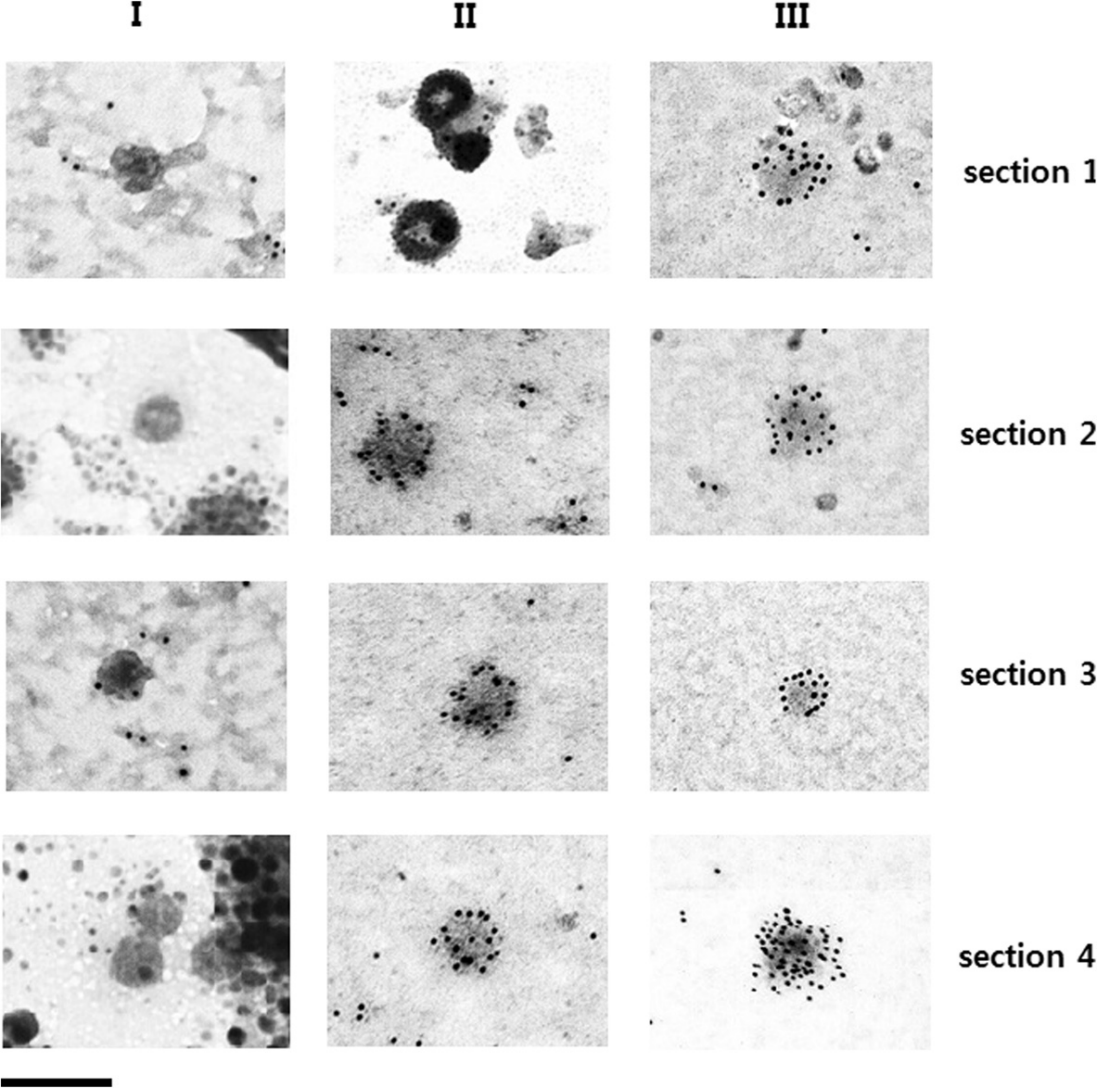
TEM analysis of HPV16 PsVs from fraction I, II and III stained with immunogold. Human histone H3 protein was detected with rabbit anti-human histone H3, together with 5 nm gold-labeled anti-rabbit IgG as described in Methods. Magnification is 234,000× (bar 100 nm)

The PsVs were negatively stained with phosphotungstic acid and observed by transmission electron microscopy (TEM) (Fig. 3 and Additional file 3: Figure S3). Fraction I PsVs appeared to be highly ordered and condensed and to be slightly smaller than those from fractions II and III (Fig. 3). Fraction II PsVs were also highly ordered but their capsomers could be seen more clearly than those of fraction I particles, indicating that they were more loosely connected. Fraction III PsVs stained strongly and appeared to consist of blackened particles (Fig. 3). Interestingly, the fraction III PsVs had a fuzzy appearance (Fig. 3 and Additional file 3: Figure S3), which we believe was due to the large amounts of associated cellular histones and DNA. When the PsVs were stained with immunogold to detect human histone H3, the surfaces of fractions II and III particles appeared to be covered by this histone (Fig. 4). The TEM images indicated that fraction I PsVs were smaller and had a more condensed structure than fractions II and III PsVs.

### Expression of reporter gene and reporter gene contents of HPV16 PsVs from fraction I, II and III

**Fig. 5:**
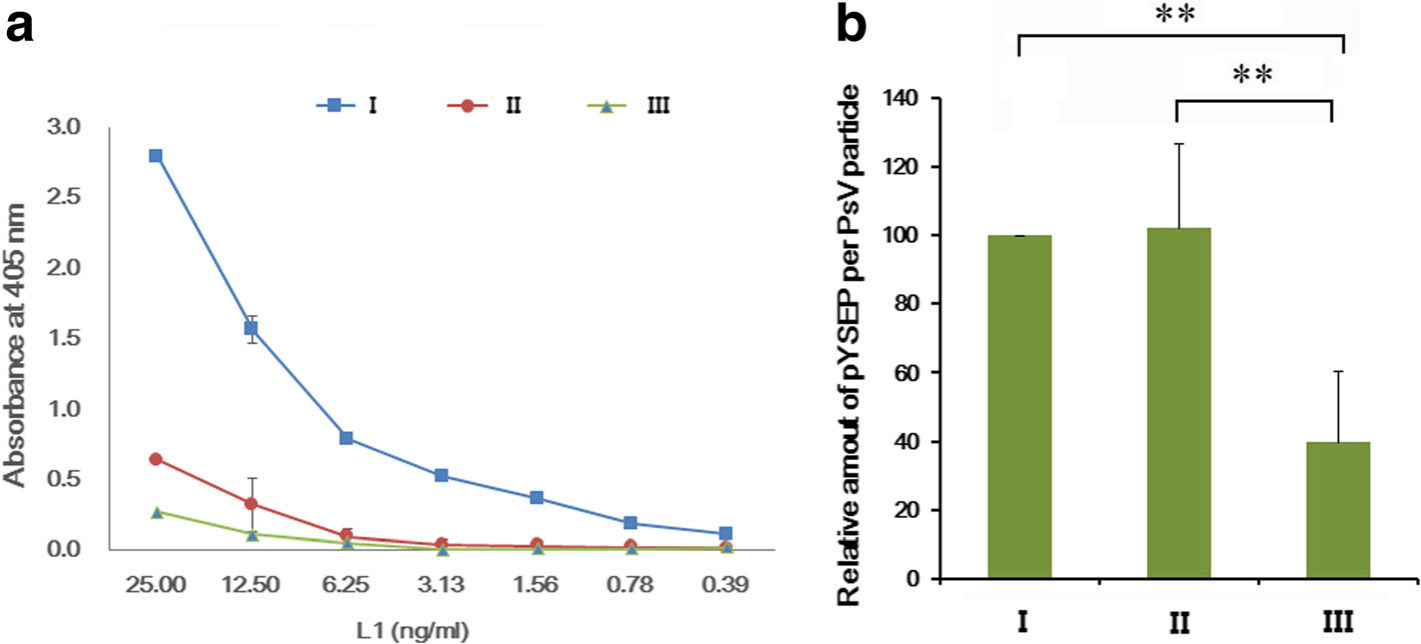
Quantification of SEAP and pYSEAP. **a** Relative SEAP expressions of PsV particles. Each type of PsV was serially diluted from 25 to 0.39 ng/mL (based on the L1 protein concentrations), and SEAP expression levels were determined as described in Methods. **b** Relative pYSEAP amounts of PsV particles. The values for fraction I PsVs in panel **b** were set at 100 %. **a** shows the mean ± SD of duplicate samples, and **b** presents the mean ± SD of four independent experiments. **, *p* < 0.01

The expression levels of reporter gene, secreted alkaline phosphatase (SEAP), in the different PsV fractions were compared (Fig. 5a). To use identical amounts of PsVs from three heparin chromatography fractions, the L1 contents were determined by sandwich ELISAs and confirmed by Western blots prior to measure the SEAP expressions. 293TT cells were plated in 96-well tissue culture plates, and each type of PsV was serially diluted from 25 ng/mL to 0.39 ng/mL and incubated for 72 h at 37 °C (Fig. 5a). Moreover, the reporter gene contents of PsVs from three fractions were determined by quantitative real-time polymerase chain reaction (PCR) (Fig. 5b). The SEAP expression levels were significantly lower in fraction II and III PsVs than in fraction I (Fig. 5a) whereas the amount of the pYSEAP reporter plasmid associated with fraction II particles appeared to be similar to that associated with fraction I particles (Fig. 5b). The amount of the pYSEAP reporter plasmid associated with fraction III particles was substantially lower (Fig. 5b). Agarose gel analysis (Additional file 4: Figure S4) supported the result of the quantitative real-time PCR in Fig. 5b. These results indicate that substantial amounts of cellular histones are detrimental for formation of PsV with high expression level of SEAP.

### The immunogenicity of HPV16 PsVs from fraction I, II and III

**Fig. 6:**
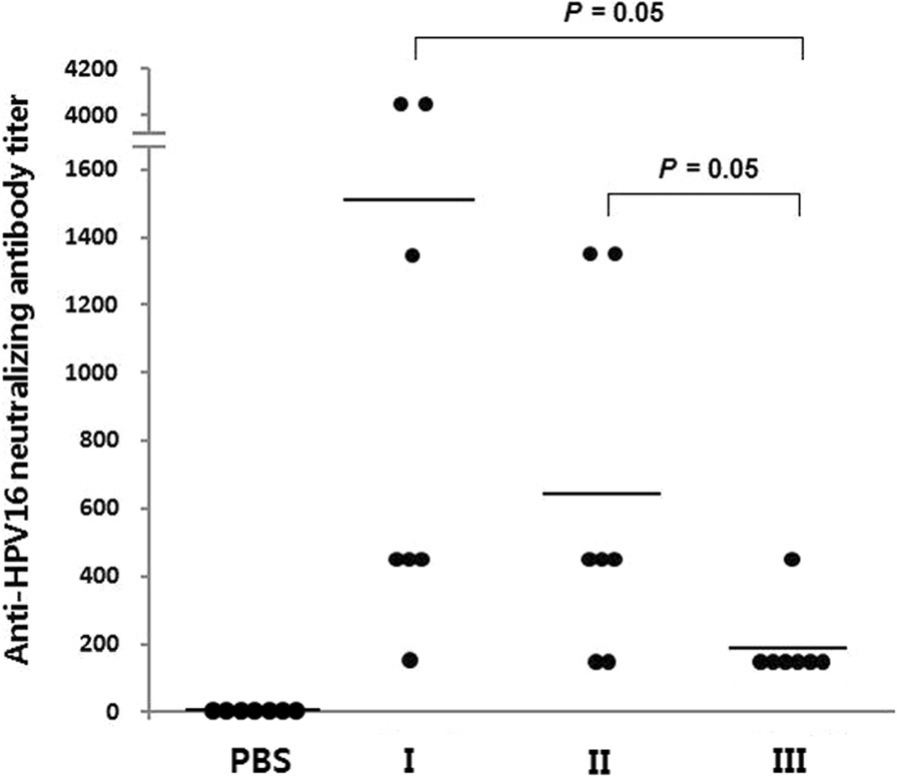
Antibody responses following three immunizations with 50 ng of PsVs per dose. Mice were immunized with PsVs (fraction I, II and III) without adjuvant. Neutralizing antibody titers against HPV16 PsVs in mouse sera were determined as described in Methods. Horizontal bars correspond to median values. PBS, *n* = 7; type I, *n* = 7; type II, *n* = 7; type III, *n* = 7

Mice were immunized three times with HPV16 PsVs, and neutralizing antibody titers were measured (Fig. 6). Fraction II and III PsVs elicited lower levels of neutralizing antibody (Fig. 6) than fraction I PsV. In addition, the mice immunized with PsVs from fraction III gave only weak lymphoproliferative responses upon stimulation with HPV16 L1 VLPs (Additional file 5: Figure S5).

## Discussion

Our results indicated that condensed structure, a substantial amount of a plasmid containing the reporter gene and a low content of cellular histones are important for the formation of infectious and immunogenic HPV16 PsVs. Of the HPV16 PsVs, 83 % appeared to be fraction I PsVs, which fulfil the requirements for highly infectious and immunogenic PsVs (Fig. 2d), whereas about 17 % of the PsVs (particles from fraction II and III) were found to have lower infectivity and immunogenicity (Fig. 2d). Therefore, it appears that most HPV16 PsV undergo assembly and maturation with high fidelity.

PsVs are known to undergo assembly within the cell, and the resulting particles are in an immature state with their capsomers only loosely connected to each other immediately after cell lysis [14]. These immature PsVs can be condensed and stabilized by a maturation process that requires incubation at 37 °C overnight [14]. It has been suggested that there are substantial losses of virus titer and of encapsidated reporter gene plasmids during the purification of immature PsV [14]. Recently, Cardone and colleagues analyzed the structures of HPV16 PsVs by time-lapse cryo-electron microscopy [33]. They reported that the diameter of the immature PsVs shrank by ∼ 5 % in the maturation process during which the interaction surfaces between adjacent capsomers were consolidated. As shown in Fig. 3 PsVs from fraction I are slightly smaller and more condensed than those from fraction II or III. In addition the capsomers of fraction II particles were less tightly connected. Infections with particles in fraction II and III led to the much lower expression of SEAP than infections with particles in fraction I (Fig. 5a). Therefore, the integrities of fraction II and III particles are similar to those of immature PsVs while the integrity of fraction I particles correspond to that of mature PsVs.

SEAP expression was significantly reduced in PsVs from fraction II (Fig. 5a) as was the elicitation of neutralizing antibodies (Fig. 6) although they harbor substantial levels of reporter gene plasmids (Fig. 5b) and have relatively satisfactory architectural structures (Fig. 3). The position of the L2 protein is known to be important for encapsidation of the reporter gene plasmid [13, 14]. However, we found that the three PsV types did not differ in terms of their L2 protein content (Additional file 6: Figure S6). In addition, ELISAs using monoclonal antibodies H16.V5 and H16.E70, which have been widely used for detecting neutralizing epitopes of the HPV16 capsid, indicated that the level of neutralizing epitopes on the surface of fraction II particles was similar to the level on the surface of fraction I particles (Additional file 7, Figure S7). Therefore, it seems that the structural robustness of fraction II PsVs is not enough for efficient delivery of the reporter gene into target cells, and its neutralizing epitopes are not recognized correctly by cells of the immune system. PsVs in fraction II were found to be covered by substantial amounts of cellular histones and cellular nucleotides (Fig. 4) and it seems likely that these interfere with binding of the PsVs to cell receptors and their correct recognition by immune cells.

Previous reports have indicated that viral DNAs containing histone, and naked DNAs without histone, coexist in native HPV preparations, and it was suggested that the DNAs of bovine papillomavirus (BPV) and HPV have a chromatin-like structure composed of nucleosomes about 8.0 nm in diameter, interconnected by naked DNA filaments [23]. Histone-free HPV DNA has been found in native HPV virions [23], and there are no histones in purified BPV [34]. Therefore, the suggestion has been made that the complex of HPV DNA with nucleoproteins is a natural intermediate during virion assembly.

The histone-containing fractions in our study were derived from HPV16 PsVs rather than native HPV virions. Our results indicated that the presence of cellular histones was detrimental to the formation of infectious PsV. However, whether the binding of histone to the surface of HPV16 PsVs shown in Fig. 4 is inherent to the HPV virion remains to be clarified. The suggested model of the assembly of HPV capsid is that the C-terminal proximal H4 helix of each L1 molecule in a capsomere invades a neighboring L1 molecule in an adjacent capsomere and forms an intermolecular disulfide bond [10]. L1 protein has a DNA binding domain at its C-terminus [35]. Therefore, there are two kinds of explanation for the effects of cellular histone. First, since immature HPV16 PsVs have greater space between neighboring capsomers (Fig. 3, see particles from fraction II) they may expose more of their DNA binding domains and so allow more binding of cellular DNA plus histones. Alternatively, the presence of cellular histones and cellular DNAs during assembly of the capsid may hinder condensation of the capsids.

## Conclusions

Our results suggest that a low content of cellular histone and cellular DNA of HPV16 PsV and a condensed structure are important for the formation of high quality HPV16 PsVs. We believe that the content of cellular histone is a critical parameter for assessing the quality and stage of maturation of HPV16 PsV. Also, the heparin chromatography technique described here appears to be a useful tool for separating PsV particles of high quality from the total pool of mature PsVs.

## Methods

### Production and purification of mature PsVs

HPV16 PsVs were produced as described by Buck and colleagues (standard protocol) [14, 36]. 293TT cells were transfected with p16sheLL (expressing L1 and L2 proteins) and pYSEAP (expressing SEAP) using Lipofectamine 2000 (Invitrogen, USA). The transfected cells were cultured for 72 h then harvested and lysed. The plasmids were kindly provided by Dr. J. T. Schiller (NIH, Bethesda, USA). To prepare a mature PsV stock, the lysate was treated with 0.1 % Benzonase (Sigma, USA) and 0.1 % Plasmid Safe (Epicentre, USA) DNase, and incubated for 24 h at 37 °C. The NaCl concentration of the lysate was adjusted to 0.8 M and clarified by centrifugation at 12,000 × *g* for 10 min at 4 °C. The PsVs in the clarified lysate were purified by SEC as follows: the clarified lysate (0.5 mL) was loaded onto a column (Tricon 10/300, 1 × 32 cm, GE Healthcare, USA) packed with Superose-6 resin (GE Healthcare, USA). The column was equilibrated with working buffer [phosphate buffered saline (PBS) + 0.52 M NaCl + 0.01 % Tween 80 pH 7.2, final NaCl concentration 0.65 M] prior to loading the sample, and the SEC was performed at a flow rate of 0.3 ml/min. Twenty fractions (0.9 mL each) were collected and analyzed by SDS-PAGE and Western blotting.

### Separation of PsVs by heparin chromatography

To separate fraction I, II and III PsVs, we modified our previous protocol for heparin chromatography [31]. A 9 cm poly-prep column (Bio-Rad, USA) was packed with 0.1 ml heparin fast-flow resin (HiPrep™ Heparin FF, GE Healthcare, USA) and equilibrated with binding buffer [PBS + 0.52 M NaCl + 0.01 % Tween 80 pH 7.2, final NaCl concentration 0.65 M]. The PsV-containing fractions from the SEC were pooled and loaded onto the heparin resin, and the capsids eluted without being bound (flow-through and wash), eluted with 0.8 M NaCl, and eluted with 1 M NaCl were considered to be fraction I, II, and III PsVs, respectively. The flow-through and eluted PsV fractions were monitored by SDS-PAGE and Western blots.

### SDS-PAGE and Western blotting

SDS-PAGE was performed according to the Laemmli’s protocol using a Mini-PROTEAN® Tetra Cell (Bio-Rad, USA). The protein bands on SDS-PAGE gels were visualized by silver staining. To detect the L1 protein by Western blotting, rabbit anti-HPV16 L1 serum and HRP-conjugated goat anti-rabbit IgG polyclonal antibody (Pierce, USA) were used [37]. Histone H3 was detected using rabbit anti-human histone H3 (sc-10809, Santa Cruz Biotechnology, USA) or rabbit anti-human histone H3 (ab1791, Abcam, USA), while histone H2B was detected with rabbit anti-human H2B (ab61250, Abcam, USA). HRP-conjugated anti-rabbit immunoglobulin G (IgG) (Bethyl, USA) was used as secondary antibody for detecting histones.

### Analysis of cellular DNA

The L1 protein content of each PsV type was determined by Western blotting and SDS-PAGE. 1 μg of L1 protein of each type was used for analyzing the content of cellular DNA. The DNA was extracted by precipitation with phenol-chloroform-isoamyl alcohol mixture (Sigma, USA), washed with 70 % ethanol and analyzed on 0.9 % agarose gels with ethidium bromide staining.

### Measurements of L1 protein amounts in HPV16 PsVs from fraction I, II, and III

To assess yields of PsVs from fraction I, II and III, their L1 protein content was determined by sandwich ELISA as previously described [32]. Purified HPV16 L1 VLPs were used as a standard. The amounts of L1 protein were confirmed by SDS-PAGE and Western blotting.

### TEM analysis

Purified PsVs (5 μg/mL) from fraction I, II and III were absorbed onto carbon-coated grids and negatively stained with 2 % phosphotungstic acid. To detect histone H3 on the surface of the PsVs, the latter were absorbed onto a carbon-coated grid, and incubated with rabbit anti-histone H3 (ab1791, Abcam, USA) followed by 5 nm gold-labeled goat anti-rabbit IgG polyclonal antibody (ab27235, Abcam, USA). Thereafter the PsVs were fixed with 1 % glutaraldehyde (Sigma, USA) and stained with 2 % uranyl acetate. Electron microscopy was performed on a TEM200CX at a final magnification of 234,000× [31].

### Determination of the level of SEAP expression per PsV particle

To determine SEAP expression levels of PsVs from fraction I, II and III, the L1 protein content of each type was determined as described above and confirmed by Western blotting. 293TT cells were plated in 96-well tissue culture plates at a density of 3 × 10^4^ cells/well for 4 h prior to PsV infection, and each type of PsV was serially diluted from 25 ng/mL to 0.39 ng/mL (based on the L1 protein concentrations) and incubated for 72 h at 37 °C. SEAP content was measured at 405 nm [31].

### Quantitative real-time PCR for pYSEAP in PsVs

To measure pYSEAP content per particle, equal amounts of PsVs from fraction I, II and III were used (200 ng based on L1 content). pYSEAP was extracted from the PsVs using a PCR extraction kit (Real Biotech Corporation, Taiwan). Prior to the extraction, the PsV stocks were treated with proteinase K (Qiagen, Germany) at 56 °C for 20 min to release encapsulated pYSEAP, and spiked with pcDNA3.1+ (Invitrogen, USA) as a reference. The primers for detecting pYSEAP were 5′-TTT AAC CAG TGC AAC ACG ACA CGC-3′ (sense) and 5′-TCC CAC TGA CTT CCC TGC TTT CTT-3′ (antisense), and those for detecting pcDNA3.1+ were 5′-ATA CGG GAT AAT ACC GCG CCA CAT-3′ (sense) and 5′-TGC ACG AGT GGG TTA CAT CGA ACT-3′ (antisense). Real-time PCR was performed using a QuantiTect SYBR Green PCR Kit (Qiagen, Germany) on an iCycler real-time PCR machine (Bio-Rad, USA). The crossing threshold (Ct) value of pYSEAP was normalized to that of pcDNA3.1+.

### Mouse immunization

Six-week-old female BALB/c mice (Orient Bio, South Korea) were assigned to four groups of seven mice each: PBS and PsVs from fraction I, II and III. The mice were subcutaneously immunized three times at two-week intervals with PsVs without adjuvant. The control group received 100 μL of PBS per dose, and the others received 50 ng of type I, II, or III PsVs per dose. Mouse sera were collected 10 days after the third immunization.

### Titrations of anti-HPV16 neutralizing antibodies from mice immunized with HPV16 PsVs

The neutralizing assays using HPV16 PsVs were performed as previously described [6, 38] The neutralization titer of a mouse serum was defined as the reciprocal of the highest dilution that caused a reduction of at least 50 % in SEAP activity [39].

### Statistical analysis

The statistical significance of differences between experimental groups was determined by two-tailed Student’s t-tests.

## Additional files

Additional file 1: Figure S1. Schematic diagram of heparin chromatography to separate HPV16 PsVs into fraction I, II and III. (DOCX 75 kb) Additional file 2: Figure S2. SDS-PAGE analysis of heparin chromatography fractions containing HPV16 PsVs. Mature HPV16 PsVs purified by SEC were further separated by heparin chromatography. The binding buffer for heparin chromatography contains 0.65 M NaCl. LS, FT and W refer to loading sample, flow-through, and wash, respectively. M indicates a protein marker. The PsVs in the flow-through and wash fractions were designated fraction I. PsVs in fraction II and III were eluted from the heparin resin by successive additions of 0.8 and 1 M NaCl. (DOCX 197 kb) Additional file 3: Figure S3. TEM analysis of HPV16 PsVs from fraction I, II and III. Magnification x 234,000 (bars are 50 or 100 nm). (DOCX 1656 kb) Additional file 4: Figure S4. PCRs for reporter genes in HPV16 PsVs from fractions I, II and III. A shows the pYSEAP contents of PsVs from fraction I, II and III. B confirms the specificity of the PCR for the relevant region of the pYSEAP construct. Buffer only and pcDNA3.1 are negative controls. To measure pYSEAP content per particle, in panel A, identical amounts of PsVs from fraction I, II, and III were used (20 ng based on L1 content). 25, 30 or 38 cycles of PCR were carried out. Thirty cycles of PCR were performed for panel B. The same primer set used in the quantitative real time PCR for amplifying the 80 bp region of pYSEAP was used (see the quantitative real time PCR section in Methods). Amplicons were analyzed on a 1.5 % agarose gel. (DOCX 406 kb) Additional file 5: Figure S5. Lymphoproliferative responses following four immunizations with HPV16 PsVs from fraction I, II, or III. The mice were immunized four times with 50 ng of PsVs per dose at 2-week intervals. Mouse splenocytes were obtained 5 days after the fourth immunization. Mouse splenocytes were labeled with carboxyfluorescein succinimidyl ester (CFSE), stimulated with purified HPV16 L1 VLPs, and cultured for 4 days. The splenocytes were stained with allophycocyanin (APC)-conjugated anti-CD4 antibody (eBioscience, USA) and examined with a FACSCalibur flow cytometer (BD Bioscience, USA). To count CD4^+^ cells, the cells were gated according to forward and side scatter, and the upper-left segment of each graph was counted on FITC and APC scatter plots. Panel A shows the flow cytometry results for three individual mice. The value in panel B represents the mean ± SEM (*n* = 3). (DOCX 171 kb) Additional file 6: Figure S6. L2 protein contents of HPV16 PsVs from fraction I, II and III. To detect the L1 and L2 proteins, 100 or 200 ng of PsVs was loaded per well (based on the L1 amount). The L2 protein was detected by Western blotting using the anti-RG1-4MAP mouse serum (1:500 dilution) with HRP conjugated goat anti-mouse IgG antibody (Bethyl Laboratories). The proteins on SDS-PAGE gels were visualized by silver staining. The quantities of the L2 protein per PsV particle were similar for all three types of PsV. (DOCX 153 kb) Additional file 7: Figure S7. Reacitivities of HPV16 PsVs from fraction I, II and III with H16.V5 or H16.E70 MAbs. The reactivities of fraction I PsVs were set at 100 %. The values are the mean ± SD of quadriplicate assays. (DOCX 43 kb)

## Abbreviations

ELISA: Enzyme-linked immunosorbent assay
GFP: Green fluorescent protein
HIV: Human immunodeficiency virus
HPV: Human papillomavirus
HPV16: Human papillomavirus type 16
IgG: Immunoglobulin G
MCV: Merkel cell polyoma virus
PsV: Pseudovirus
QV: Quasivirions
SDS-PAGE: Sodium dodecyl sulfate-polyacrylamide gel electrophoresis
SEAP: Secreted alkaline phosphatase
SEC: Size-exclusion chromatography
SV40: Simian virus 40
TEM: Transmission electron microscopy
VLP: Virus-like particle
