# Supplementary material for: Characterization of human papillomavirus type 16 pseudovirus containing histones
Source: BMC Biotechnol. 2016 Aug 27;16(1):63. doi: 10.1186/s12896-016-0296-3 (PMC5002194; doi:10.1186/s12896-016-0296-3)

Additional file 1: Fig. S1. Schematic diagram of heparin chromatography to separate HPV16 PsVs into fraction I, II and III


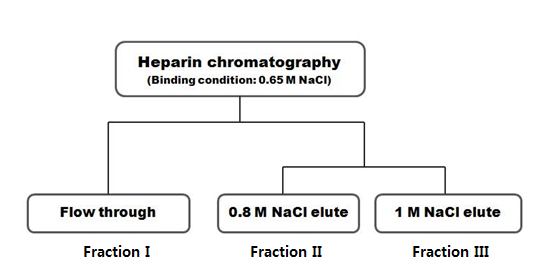

Supplement: Additional file 1: Figure S1. — Schematic diagram of heparin chromatography to separate HPV16 PsVs into fraction I, II and III. (DOCX 75 kb) [file 12896_2016_296_MOESM1_ESM.docx]
