# Supplementary material for: Characterization of human papillomavirus type 16 pseudovirus containing histones
Source: BMC Biotechnol. 2016 Aug 27;16(1):63. doi: 10.1186/s12896-016-0296-3 (PMC5002194; doi:10.1186/s12896-016-0296-3)

Additional file 2: Fig. S2. SDS-PAGE analysis of heparin chromatography fractions containing HPV16 PsVs. Mature HPV16 PsVs purified by SEC were further separated by heparin chromatography. The binding buffer for heparin chromatography contains 0.65 M NaCl. LS, FT and W refer to loading sample, flow-through, and wash, respectively. M indicates a protein marker. The PsVs in the flow-through and wash fractions were designated fraction I. PsVs in fraction II and III were eluted from the heparin resin by successive additions of 0.8 and 1 M NaCl.


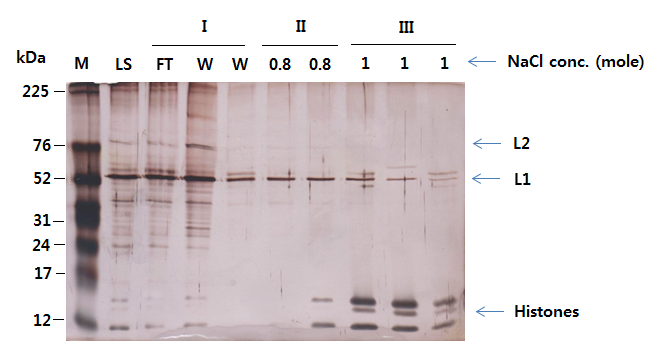

Supplement: Additional file 2: Figure S2. — SDS-PAGE analysis of heparin chromatography fractions containing HPV16 PsVs. Mature HPV16 PsVs purified by SEC were further separated by heparin chromatography. The binding buffer for heparin chromatography contains 0.65 M NaCl. LS, FT and W refer to loading sample, flow-through, and wash, respectively. M indicates a protein marker. The PsVs in the flow-through and wash fractions were designated fraction I. PsVs in fraction II and III were eluted from the heparin resin by successive additions of 0.8 and 1 M NaCl. (DOCX 197 kb) [file 12896_2016_296_MOESM2_ESM.docx]
