# Supplementary material for: Characterization of human papillomavirus type 16 pseudovirus containing histones
Source: BMC Biotechnol. 2016 Aug 27;16(1):63. doi: 10.1186/s12896-016-0296-3 (PMC5002194; doi:10.1186/s12896-016-0296-3)

Additional file 3: Fig. S3. TEM analysis of HPV16 PsVs from fraction I, II and III. Magnification x 234,000 (bars are 50 or 100 nm).


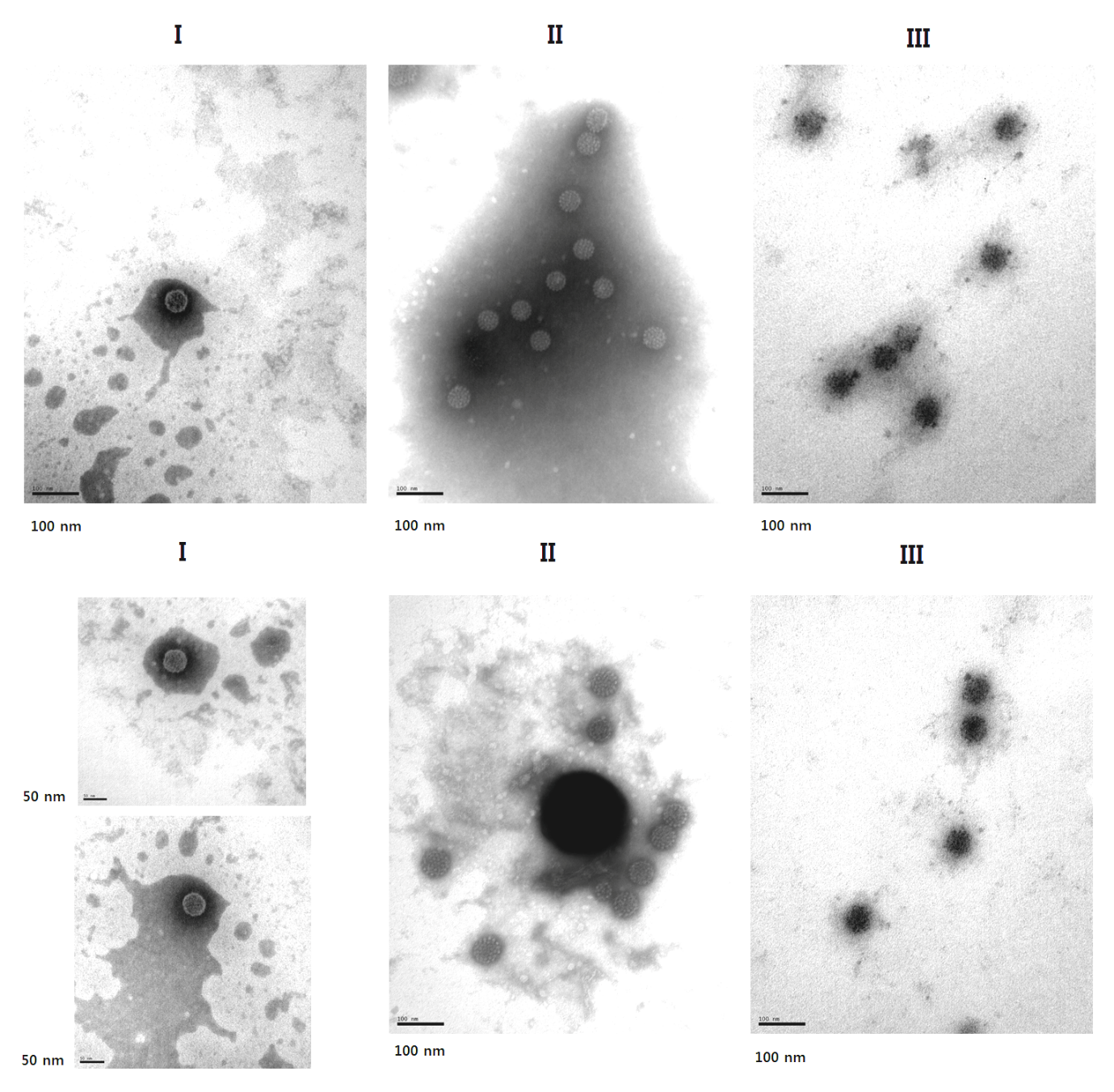

Supplement: Additional file 3: Figure S3. — TEM analysis of HPV16 PsVs from fraction I, II and III. Magnification x 234,000 (bars are 50 or 100 nm). (DOCX 1656 kb) [file 12896_2016_296_MOESM3_ESM.docx]
