# Supplementary material for: Characterization of human papillomavirus type 16 pseudovirus containing histones
Source: BMC Biotechnol. 2016 Aug 27;16(1):63. doi: 10.1186/s12896-016-0296-3 (PMC5002194; doi:10.1186/s12896-016-0296-3)

Attachment file 4: Fig. S4. PCRs for reporter genes in HPV16 PsVs from fractions I, II and III. A shows the pYSEAP contents of PsVs from fraction I, II and III. B confirms the specificity of the PCR for the relevant region of the pYSEAP construct. Buffer only and pcDNA3.1 are negative controls. To measure pYSEAP content per particle, in panel A, identical amounts of PsVs from fraction I, II, and III were used (20 ng based on L1 content). 25, 30 or 38 cycles of PCR were carried out. Thirty cycles of PCR were performed for panel B. The same primer set used in the quantitative real time PCR for amplifying the 80 bp region of pYSEAP was used (see the quantitative real time PCR section in Methods). Amplicons were analyzed on a 1.5% agarose gel.


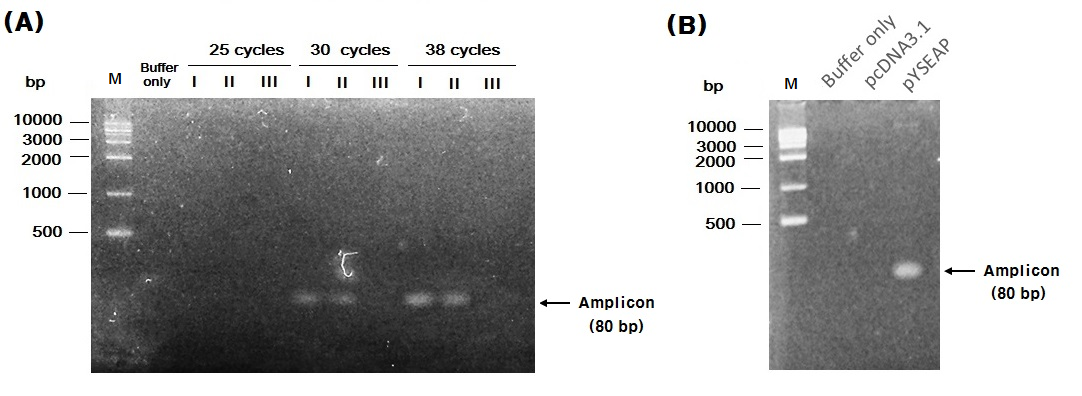

Supplement: Additional file 4: Figure S4. — PCRs for reporter genes in HPV16 PsVs from fractions I, II and III. A shows the pYSEAP contents of PsVs from fraction I, II and III. B confirms the specificity of the PCR for the relevant region of the pYSEAP construct. Buffer only and pcDNA3.1 are negative controls. To measure pYSEAP content per particle, in panel A, identical amounts of PsVs from fraction I, II, and III were used (20 ng based on L1 content). 25, 30 or 38 cycles of PCR were carried out. Thirty cycles of PCR were performed for panel B. The same primer set used in the quantitative real time PCR for amplifying the 80 bp region of pYSEAP was used (see the quantitative real time PCR section in Methods). Amplicons were analyzed on a 1.5 % agarose gel. (DOCX 406 kb) [file 12896_2016_296_MOESM4_ESM.docx]
