# Supplementary material for: Characterization of human papillomavirus type 16 pseudovirus containing histones
Source: BMC Biotechnol. 2016 Aug 27;16(1):63. doi: 10.1186/s12896-016-0296-3 (PMC5002194; doi:10.1186/s12896-016-0296-3)

Additional file 5: Fig. S5. Lymphoproliferative responses following four immunizations with HPV16 PsVs from fraction I, II, or III. The mice were immunized four times with 50 ng of PsVs per dose at two-week intervals. Mouse splenocytes were obtained 5 days after the fourth immunization. Mouse splenocytes were labeled with carboxyfluorescein succinimidyl ester (CFSE), stimulated with purified HPV16 L1 VLPs, and cultured for 4 days. The splenocytes were stained with allophycocyanin (APC)-conjugated anti-CD4 antibody (eBioscience, USA) and examined with a FACSCalibur flow cytometer (BD Bioscience, USA). To count CD4^+^ cells, the cells were gated according to forward and side scatter, and the upper-left segment of each graph was counted on FITC and APC scatter plots. Panel A shows the flow cytometry results for three individual mice. The value in panel B represents the mean ± SEM (n=3).


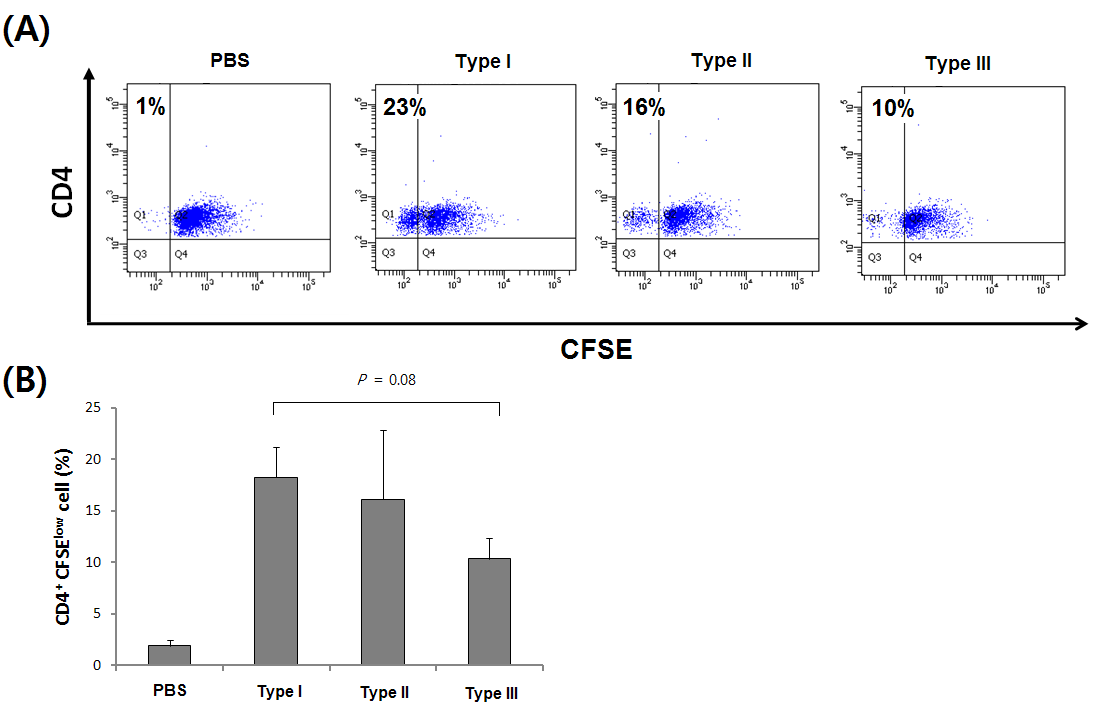

Supplement: Additional file 5: Figure S5. — Lymphoproliferative responses following four immunizations with HPV16 PsVs from fraction I, II, or III. The mice were immunized four times with 50 ng of PsVs per dose at 2-week intervals. Mouse splenocytes were obtained 5 days after the fourth immunization. Mouse splenocytes were labeled with carboxyfluorescein succinimidyl ester (CFSE), stimulated with purified HPV16 L1 VLPs, and cultured for 4 days. The splenocytes were stained with allophycocyanin (APC)-conjugated anti-CD4 antibody (eBioscience, USA) and examined with a FACSCalibur flow cytometer (BD Bioscience, USA). To count CD4+ cells, the cells were gated according to forward and side scatter, and the upper-left segment of each graph was counted on FITC and APC scatter plots. Panel A shows the flow cytometry results for three individual mice. The value in panel B represents the mean ± SEM (n = 3). (DOCX 171 kb) [file 12896_2016_296_MOESM5_ESM.docx]
