# Supplementary material for: Characterization of human papillomavirus type 16 pseudovirus containing histones
Source: BMC Biotechnol. 2016 Aug 27;16(1):63. doi: 10.1186/s12896-016-0296-3 (PMC5002194; doi:10.1186/s12896-016-0296-3)
Supplement: Additional file 6: Figure S6. — L2 protein contents of HPV16 PsVs from fraction I, II and III. To detect the L1 and L2 proteins, 100 or 200 ng of PsVs was loaded per well (based on the L1 amount). The L2 protein was detected by Western blotting using the anti-RG1-4MAP mouse serum (1:500 dilution) with HRP conjugated goat anti-mouse IgG antibody (Bethyl Laboratories). The proteins on SDS-PAGE gels were visualized by silver staining. The quantities of the L2 protein per PsV particle were similar for all three types of PsV. (DOCX 153 kb) [file 12896_2016_296_MOESM6_ESM.docx]

Additional file 6: Fig. S6. L2 protein contents of HPV16 PsVs from fraction I, II and III. To detect the L1 and L2 proteins, 100 or 200 ng of PsVs was loaded per well (based on the L1 amount). The L2 protein was detected by Western blotting using the anti-RG1-4MAP mouse serum (1:500 dilution) with HRP conjugated goat anti-mouse IgG antibody (Bethyl Laboratories). The proteins on SDS-PAGE gels were visualized by silver staining. The quantities of the L2 protein per PsV particle were similar for all three types of PsV.


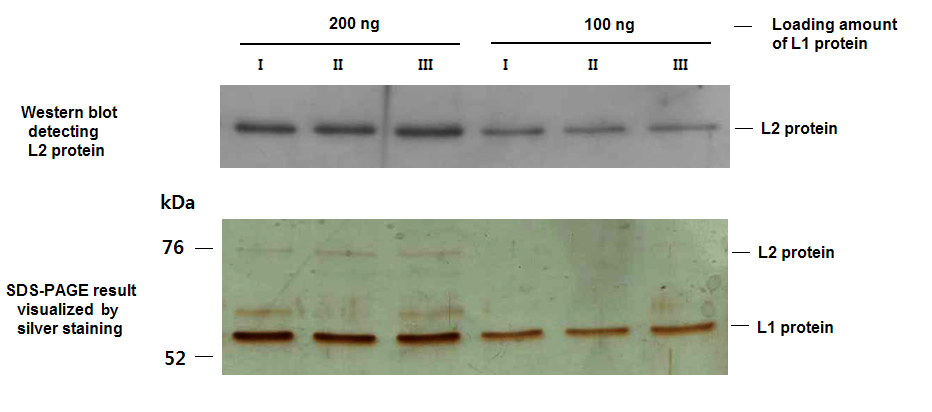


**Preparation of mouse antibody against RG1 residue of L2 protein**

RG1-4MAP was synthesized to obtain antibodies reacting with the HPV16 L2 protein. The amino acid sequence 17–36 (RG1) of the L2 minor capsid protein [[Reference](#_ENREF_1)] was synthesized, and the C-terminus was linked to 4-branch multiple antigenic peptides (4MAP). The RG1 sequence was QLYKTCKQAGTCPPDIIPKV-4MAP. The mice were immunized subcutaneously three times with 200 µg RG1-4MAP combined with Freund’s adjuvant, and mouse serum was obtained from a tail vein 10 days after the third immunization.

Reference

Alphs HH, Gambhira R, Karanam B, Roberts JN, Jagu S, Schiller JT, Zeng W, Jackson DC, Roden RB: **Protection against heterologous human papillomavirus challenge by a synthetic lipopeptide vaccine containing a broadly cross-neutralizing epitope of L2**. *Proc Natl Acad Sci U S A* 2008, **105**(15):5850-5855.
