# Supplementary material for: Characterization of human papillomavirus type 16 pseudovirus containing histones
Source: BMC Biotechnol. 2016 Aug 27;16(1):63. doi: 10.1186/s12896-016-0296-3 (PMC5002194; doi:10.1186/s12896-016-0296-3)

Additional file 7: Fig. S7. Reacitivities of HPV16 PsVs from fraction I, II and III with H16.V5 or H16.E70 MAbs. The reactivities of fraction I PsVs were set at 100%. The values are the mean ± SD of quadriplicate assays.

**Enzyme-linked immunosorbent assay (ELISA) to detect neutralizing epitopes on PsVs**

Monoclonal antibodies (MAbs) H16.V5 and H16.E70 were used to detect neutralizing epitopes on the PsVs. Mabs were kindly provided by Dr. N. D. Christensen (Pennsylvania State University College of Medicine, USA). A 96-well ELISA plate (Greiner Bio One, Germany) was coated with 100 ng of H16.V5 or H16.E70 per well and blocked with 5% skim milk in PBS containing 0.05% Tween 20 (PBST). The plate was incubated with 500 ng/mL of fraction I, II, or III PsVs at 37°C for 2 h. VLPs bound to MAbs were detected using rabbit anti-HPV16 L1 polyclonal antibody and HRP-conjugated goat anti-rabbit IgG antibody (Bethyl Laboratories, USA). Color reactions were developed with *o*-phenylenediamine (Sigma, USA), and optical density was measured at 492 nm.


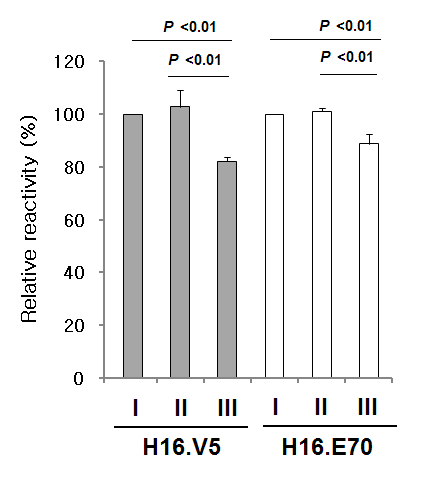

Supplement: Additional file 7: Figure S7. — Reacitivities of HPV16 PsVs from fraction I, II and III with H16.V5 or H16.E70 MAbs. The reactivities of fraction I PsVs were set at 100 %. The values are the mean ± SD of quadriplicate assays. (DOCX 43 kb) [file 12896_2016_296_MOESM7_ESM.docx]
